# Persistent lactic acidosis in ALK-positive anaplastic large cell lymphoma: a case report and literature review

**DOI:** 10.3389/fonc.2026.1744202

**Published:** 2026-02-27

**Authors:** Haoru Jin, Zhibin Xu, Qing Ai, Qiangwei Huang, Xu Chen, Yongbei Luo, Peicong Hong, Yuan Qiu

**Affiliations:** 1 Department of Thoracic Surgery, The First Affiliated Hospital of Guangzhou Medical University, Guangzhou, Guangdong, China; 2 Department of Organ Tansplantation, Guangzhou Institute of Respiratory Health, Guangzhou, Guangdong, China; 3 Department of Surgery, National Center for Respiratory Medicine, Guangzhou, Guangdong, China; 4 Department of Surgery, National Clinical Research Center for Respiratory Disease, Guangzhou, Guangdong, China; 5 Department of Surgery, State Key Laboratory of Respiratory Disease, Guangzhou, Guangdong, China; 6 Department of Organ Tansplantation, The First Affiliated Hospital of Guangzhou Medical University, Guangzhou, Guangdong, China; 7 Department of Surgery, Guangzhou Chest Hospital, Guangzhou, Guangdong, China

**Keywords:** ALK-positive anaplastic large cell lymphoma, chemotherapy response, tumor metabolism, type B lactic acidosis, Warburg effect

## Abstract

Lactic acidosis is common in the ICU, but malignancy-associated type B lactic acidosis mediated by the clinical Warburg effect (CWE) is uncommon and easily missed when infection or organ dysfunction dominates the presentation. We report a 35-year-old man with a month of fever and progressive dyspnea who presented with persistent hyperlactatemia (peak 15.46 mmol/L) and markedly elevated LDH despite stable hemodynamics. Imaging revealed generalized lymphadenopathy, multifocal osteolytic lesions, and bilateral pleural effusions; infectious studies were unrevealing apart from chronic hepatitis B. Cervical node biopsy (CD30^+^, ALK-L^+^, EMA^+^; Ki-67 ~80%; EBER–; pan-B/T and epithelial markers negative) established ALK-positive anaplastic large-cell lymphoma. In the absence of shock or sustained hypoperfusion, CWE/type B lactic acidosis was diagnosed. An etoposide-containing CHOP variant (ECHOP/CHOEP, days 1–5) was initiated with parallel continuous renal replacement therapy (CVVHDF) to stabilize acid–base and electrolytes as a bridge to chemotherapy. Lactate declined from 13.30 mmol/L immediately before chemotherapy to 2.88 mmol/L by day 3, 1.33 mmol/L by day 8, and normalized (0.6 mmol/L) by day 17, closely tracking clinical improvement. The patient was extubated, CRRT discontinued, transferred out of the ICU, and discharged; chemotherapy-related myelosuppression and ICU-acquired weakness were managed with G-CSF, transfusions, and early rehabilitation, and a femoral deep-vein thrombosis was treated with anticoagulation. A focused review of 18 recent case reports (2021–2025) suggests that timely, standardized antitumor therapy is frequently followed by a rapid, time-locked fall in lactate, whereas the absence of definitive oncologic treatment portends uniformly poor short-term outcomes. This case underscores that, in hemodynamically stable patients with refractory hyperlactatemia, early consideration of CWE and prompt tumor-directed therapy—supported by targeted organ support such as CRRT—offer the most reliable path to metabolic reversal and recovery.

## 1 Introduction

Lactic acidosis ranks among the most common metabolic disturbances in critical care settings. Initial assessment should prioritize identifying and addressing impaired perfusion or oxygen delivery; only after these are stabilized should alternative etiologies be explored (1, 2). Drawing on the classic Cohen-Woods classification and its modern applications, lactic acidosis is categorized as type A (primarily linked to shock, hypovolemia, severe hypoxemia, or mitochondrial toxins that induce tissue hypoxia) or type B (arising without evident hypoperfusion and stemming from metabolic disorders, drugs or toxins, impaired hepatic or renal clearance, or malignancy) (3, 4). In clinical practice, this distinction is rarely absolute: multiple mechanisms may overlap in a single patient, underscoring the need for early detection—and ongoing reevaluation—of any evolving hypoperfusion to ensure precise etiologic diagnosis.

Epidemiologically, hyperlactatemia is prevalent in intensive care unit (ICU) populations, yet Warburg effect-mediated type B lactic acidosis remains rare and can be masked by concurrent infections, organ failure, or tumor necrosis, often leading to delayed diagnosis. Once progressive hypoperfusion and hypoxemia have been reasonably ruled out, persistent hyperlactatemia with markedly elevated lactate dehydrogenase (LDH) levels and minimal response to intensified antimicrobials or organ support should raise suspicion for malignancy-associated type B lactic acidosis (5). For instance, Nakagawa et al. (6) reported anaplastic lymphoma kinase (ALK)-positive anaplastic large-cell lymphoma (ALCL) presenting with lactic acidosis in the absence of shock, where lactate levels normalized rapidly following EPOCH chemotherapy. Similarly, Wang et al. (7) described type B lactic acidosis accompanied by hypoglycemia in diffuse large B-cell lymphoma (DLBCL)—a hallmark of the Warburg effect—in which targeted chemotherapy was instrumental in resolution.

Mechanistically, this presentation aligns with the Warburg effect, wherein tumor cells favor aerobic glycolysis even in oxygen-replete conditions, diverting substantial glucose to lactate production to fuel biosynthetic needs and maintain redox homeostasis amid rapid proliferation—a metabolic hallmark of cancer (8, 9). Emerging evidence suggests that lactate is far from a mere byproduct; it serves as an active signaling molecule and epigenetic regulator that remodels the tumor microenvironment, dampens antitumor immunity, and modulates therapeutic responses (10, 11). Thus, sustained hyperlactatemia may act both as a marker of metabolic dysregulation and as a contributor to disease advancement.

Clinically, the Warburg effect manifests at the bedside as profound, refractory hyperlactatemia without overt shock or persistent hypoperfusion, frequently correlating with greater tumor burden, bone involvement, and poorer prognosis (12). In observational cohorts, tumor-directed therapies have often elicited a swift lactate decline—a “metabolic response” temporally aligned with treatment initiation—highlighting the critical role of addressing the underlying malignancy in resolving the metabolic derangement (13, 14). Nonetheless, interpretive caution is essential: coexisting hepatic or renal dysfunction, drug or toxin exposures, infections, and perfusion variability can confound assessments, and clinicians must refrain from attributing elevated lactate solely to the Warburg effect without a comprehensive differential diagnosis.

Building on these insights, we report a case of a critically ill patient presenting with persistent hyperlactatemia as the predominant feature, ultimately confirmed to have aggressive lymphoma and exhibiting a rapid metabolic response—manifested as a swift decline in lactate levels—following chemotherapy initiation. Although imaging findings and elevated LDH levels supported early suspicion of malignant lymphoma and a Warburg effect–related mechanism, this case represents a typical and well-documented clinical presentation of tumor-associated type B lactic acidosis. By clearly illustrating the metabolic trajectory and treatment response, this report provides a practical diagnostic and therapeutic framework for clinicians. Furthermore, it underscores the value of timely multidisciplinary management integrating organ support and definitive chemotherapy.

## 2 Case presentation

### 2.1 Patient information

A 35-year-old man presented to our institution on June 3, 2025, with recurrent fevers for more than 1 month, which had worsened with accompanying dyspnea over the preceding 2 weeks. Past medical history was notable for chronic hepatitis B infection with irregular antiviral therapy, a 15-pack-year smoking history (20 cigarettes per day), and a history of methamphetamine use by inhalation. He had no known history of hypertension, diabetes mellitus, or other chronic comorbidities; no prior surgeries or trauma; and no reported drug allergies.

The patient’s symptoms began in May 2025 following exposure to rain and cold, manifestingas fever with a maximum temperature of 39.6 °C. He was initially evaluated at local community hospitals, where he was diagnosed with mycoplasma infection and treated with moxifloxacin, without symptomatic improvement. He was subsequently transferred to a regional tertiary referral center from May 13 to 17, where he received meropenem combined with doxycycline for empiric antimicrobial coverage; however, fevers persisted, and dyspnea progressively intensified. He was transferred again and admitted to another municipal tertiary care hospital from May 22 to June 3. There, chest computed tomography (CT) and magnetic resonance imaging (MRI) demonstrated diffuse multifocal bone lesions throughout the skeleton. Bone marrow biopsy was planned to establish a definitive diagnosis, but due to escalating dyspnea and lethargy, the patient and his family elected for discharge against medical advice, prompting referral to our hospital(**Supplementary Figure 1**).

### 2.2 Physical examination

On admission, vital signs were notable for a temperature of 38 °C, heart rate of 165 beats per minute, respiratory rate of 31 breaths per minute, blood pressure of 161/68 mm Hg, and oxygen saturation of 90% on room air, with no hypotension or evidence of shock. The patient appeared acutely ill, with lethargy but appropriate responses to questions and fair cooperation during examination. Physical findings included scattered petechiae, ecchymoses, and reddish-brown nodules on the skin and mucous membranes; subcutaneous nodules were palpable on the scalp. Multiple enlarged lymph nodes were noted bilaterally in the cervical, supraclavicular, axillary, and inguinal regions, with the largest measuring approximately 41 × 19 mm; these were firm, poorly mobile, and nontender. Respiratory examination revealed coarse breath sounds bilaterally, with wet crackles in the lower lung fields, no pleural rubs, and marked dyspnea on exertion. Cardiovascular assessment showed tachycardia at 165 beats per minute with a regular rhythm and no pathologic murmurs auscultated over the valvular areas. The abdomen was soft and nontender, without rebound tenderness; the liver and spleen were not enlarged, and shifting dullness was absent. Neurologic evaluation was limited by poor cooperation for muscle strength testing, but physiologic reflexes were present, and pathologic reflexes were not elicited. He denied chest pain or hemoptysis, abdominal pain or diarrhea, and urinary frequency, urgency, or dysuria; he reported a weight loss of approximately 5 kg over the past month.

### 2.3 Laboratory & imaging findings

#### 2.3.1 Metabolic and organ function parameters

Lactate levels were markedly elevated on admission (June 3, 2025: 10.92 mmol/L; reference range: 0.5-2.2 mmol/L), peaking at 15.46 mmol/L on June 4, declining to 2.88 mmol/L following chemotherapy initiation on June 13, and normalizing to 0.6 mmol/L by June 27.

Liver and kidney function tests on June 4 showed alanine aminotransferase (ALT) at 347.0 U/L (elevated), total bilirubin at 33.1 μmol/L (elevated), and albumin at 26.3 g/L (decreased). By June 30, creatinine was 27.30 μmol/L (reference range: 44-133 μmol/L), consistent with an early compensatory phase of renal function.

Electrolyte panel revealed hypercalcemia on June 3 (serum calcium: 2.85 mmol/L; elevated), hypomagnesemia on June 17 (serum magnesium: 0.54 mmol/L; decreased), with serum calcium normalizing to 2.13 mmol/L by June 30.

Cardiac biomarkers included lactate dehydrogenase (LDH) at 2063.0 U/L (elevated) on June 3, which decreased to 327.0 U/L (still elevated) by July 3.

Arterial blood gas showed pH 7.30 and pCO_2_ 31 mmHg at admission.

#### 2.3.2 Infection-related parameters

Procalcitonin (PCT) was elevated at 3.679 ng/mL on June 4, decreasing to 0.244 ng/mL (still elevated) by July 6.

Pathogen evaluations were negative for sputum acid-fast bacilli smear and Mycobacterium tuberculosis DNA (TB-DNA). Throat swabs were negative for influenza A/B, respiratory syncytial virus, and SARS-CoV-2 nucleic acid amplification tests. Metagenomic next-generation sequencing (mNGS) of bronchoalveolar lavage fluid detected only hepatitis B virus, while fungal culture grew Candida glabrata (sensitive to fluconazole and voriconazole).

#### 2.3.3 Imaging studies

**Figure 1:**
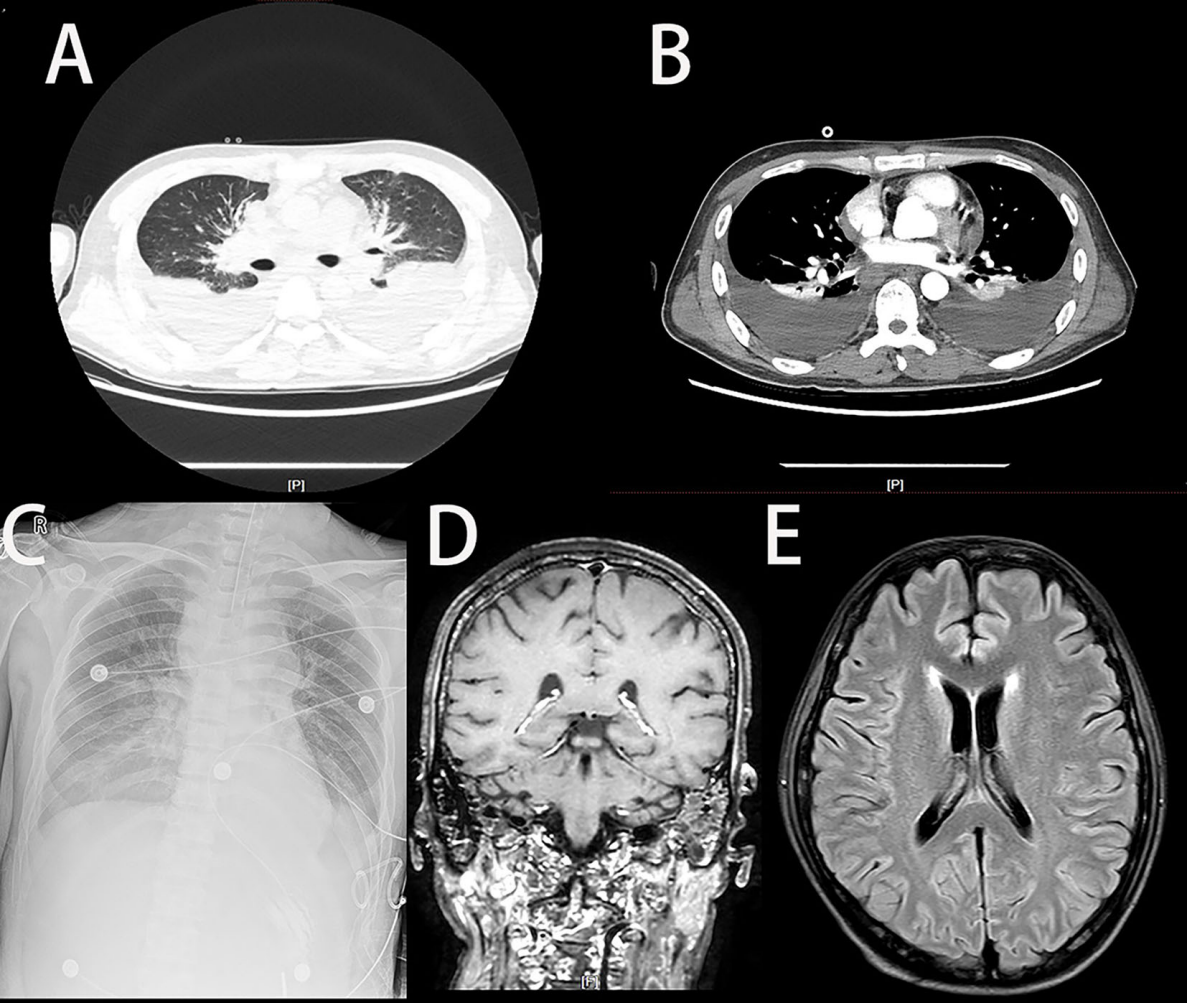
Admission imaging findings. Radiologic studies upon admission showed pleural effusion and pulmonary exudates, consistent with infection. **(A)** Axial non-contrast chest CT (lung window). **(B)** Axial non-contrast chest CT (mediastinal window). **(C)** Posteroanterior (PA) chest radiograph. **(D)** Coronal brain MRI. **(E)** Axial brain MRI (FLAIR sequence).

Contrast-enhanced chest CT (June 3) demonstrated bilateral cervical, mediastinal, and hilar lymphadenopathy; multifocal osteolytic lesions involving the right first rib and thoracolumbar vertebrae; and bilateral moderate pleural effusions (**Figure 1A**).

Lymph node ultrasonography (June 4) revealed abnormal architecture in the left inguinal lymph node and bilateral cervical lymph nodes, suggestive of malignancy (**Figure 1B**).

Transthoracic echocardiography (mid-June) showed mild tricuspid regurgitation, mild pulmonary hypertension, a small pericardial effusion, and preserved left ventricular systolic function (**Figure 1C**).

Non-contrast and contrast-enhanced brain MRI (late June) identified bilateral sphenoid and left frontal sinusitis, as well as bilateral mastoiditis, with no evidence of metastatic disease (**Figure 1D**).

#### 2.3.4 Pathologic

**Figure 2:**
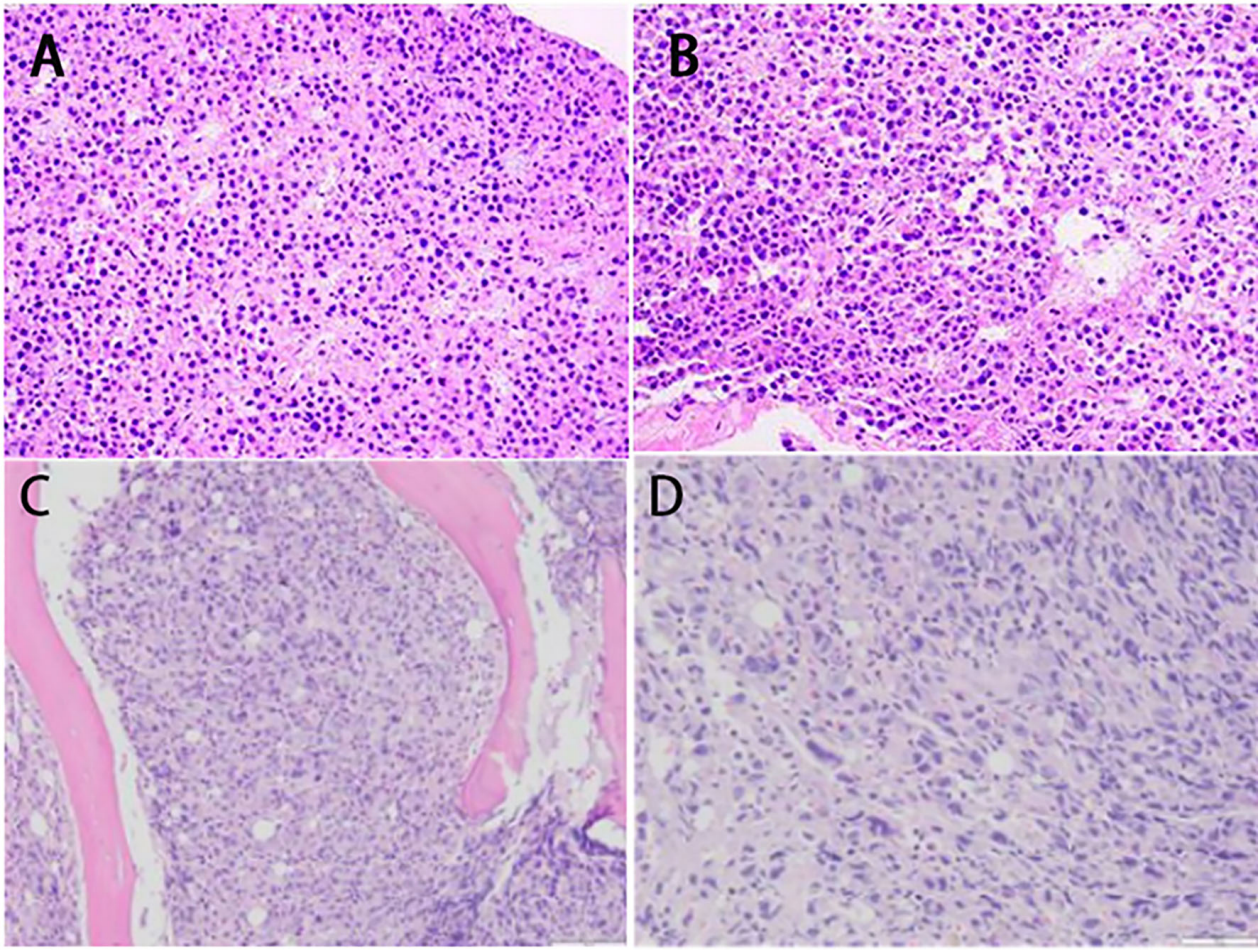
The morphological manifestations of right cervical lymph node puncture/biopsy and bone tissue biopsy. **(A)** Under low magnification (×100), the tumor cells show dense, sheet-like growth with diffuse infiltration of submitted fibrous and striated muscle tissue. **(B)** Under high magnification (×200), the tumor cells appear oval, reniform, or irregular in shape, with visible mitotic figures and eosinophilic cytoplasm—features consistent with anaplastic large cell lymphoma. Combined with the immunohistochemical profile (CD30^+^, ALK-L^+^, Ki-67 ≈ 80%^+^, EBER^-^), the final diagnosis was.ALK-positive anaplastic large cell lymphoma (ALK^+^ ALCL). Immunohistochemical staining demonstrates the following profile: CD30^+^, ALK-L^+^, Ki-67 ≈ 80%^+^, EMA^+^, EBER^-^, CD3 (scattered^+^), CD5 (partial^+^), CD2^+^, Mum-1^+^, P53 (wild-type expression), and negative for CD20, CD21, CD23, CD38, CD56, CD68, CD117, CD34, and MPO. **(C)** Under low magnification (×100), tumor cells diffusely infiltrate the bone marrow tissue, showing a clear demarcation from the surrounding bony trabeculae (pink areas) and markedly increased cellular density. **(D)** Under high magnification (×200), the tumor cells appear oval or irregular in shape with pleomorphic nuclei and abundant cytoplasm, consistent with the cytomorphologic features of anaplastic large cell lymphoma, indicating infiltrative growth of the tumor within the bone marrow.

On June 5, needle biopsy of an enlarged right cervical lymph node was performed (**Figures 2A, B**). Consultation on bone marrow slides from an outside institution on June 6 confirmed the diagnosis of ALK-positive anaplastic large cell lymphoma. Immunohistochemistry demonstrated CD30 positivity, ALK-L positivity, a Ki-67 proliferation index of 80%, and negative Epstein-Barr virus-encoded RNA (EBER) (**Figures 2C, D**).

## 3 Diagnostic assessment

- Sepsis
- Lactic acidosis
- ALK-positive anaplastic large cell lymphoma
- ICU-acquired weakness
- Chronic moderate hepatitis B

## 4 Clinical course & follow-up

### 4.1 Initial supportive care phase (june 3 to june 9)

Following admission, due to worsening dyspnea and oxygen saturation declining to 90%, the patient underwent endotracheal intubation via the nasal route and was placed on mechanical ventilation (synchronized intermittent mandatory ventilation [SIMV] mode, FiO_2_ 60%) for respiratory support. Empiric antimicrobial therapy was initiated with piperacillin-tazobactam (Tazocin^®^, Wyeth/Pfizer). On June 4, suspecting Penicillium marneffei infection, the regimen was adjusted to meropenem (Merrem^®^, Sumitomo Pharma Co., Ltd.) combined with liposomal amphotericin B (AmBisome^®^, Gilead Sciences, Inc.); liposomal amphotericin B was discontinued on June 6 after outside institution bone marrow next-generation sequencing (NGS) yielded no fungal detection.

Symptomatic management included entecavir (Baraclude^®^, Bristol-Myers Squibb) for antiviral therapy against hepatitis B (admission HBV-DNA: 1.43 × 10^5^ IU/mL), glutathione for hepatoprotection, and recombinant human thrombopoietin injection (TPIAO^®^, Shenyang Sunshine Pharmaceutical Co., Ltd.) to elevate platelet count (June 4: 53 × 10^9^/L). On June 6, left-sided thoracentesis and drainage were performed to alleviate compressive symptoms from pleural effusion. On June 8, the patient developed paroxysmal rapid atrial fibrillation, managed with intravenous esmolol hydrochloride (Ailuo^®^, Qilu Pharmaceutical Co., Ltd.) infusion and oral amiodarone (Cordarone^®^, Sanofi) for ventricular rate control.

### 4.2 Core therapeutic phase (june 10 to june 24)

Following confirmation of ALK-positive ALCL on June 10, antineoplastic therapy was initiated with the ECHOP chemotherapy regimen: etoposide (Vepesid^®^, Bristol-Myers Squibb) 50 mg on days 1-5, cyclophosphamide (Endoxan^®^, Baxter Oncology GmbH) 1 g on day 1, vindesine (Hangzhou Minsheng Pharmaceutical Co., Ltd.) 4 mg on day 1, epirubicin (Farmorubicin^®^, Pfizer Pharmaceutical Co., Ltd.) 90 mg on day 1, and methylprednisolone (Solu-Medrol^®^, Pfizer Manufacturing Belgium NV) 40 mg on days 1-5. The first cycle was completed on June 13.

Additionally, on June 10, in response to acute kidney injury (manifested by oliguria) and refractory metabolic acidosis (pH 7.313, bicarbonate 14.5 mmol/L), continuous renal replacement therapy (CRRT) was commenced (continuous venovenous hemodiafiltration [CVVHDF] mode with bicarbonate replacement fluid); this was discontinued on June 18 following improvement in renal function.

On June 11, antimicrobial therapy was adjusted to include vancomycin (Zhejiang Medicine Co., Ltd.) for gram-positive coverage after sputum smear revealed occasional gram-positive cocci; vancomycin was discontinued on June 23 as inflammatory markers declined. By June 24, antibiotics were de-escalated to piperacillin-tazobactam (Tazocin^®^, Wyeth/Pfizer), with continuation of voriconazole (VFEND^®^, Pfizer Pharmaceutical Co., Ltd.) for antifungal prophylaxis (based on bronchoalveolar lavage fluid culture yielding Candida glabrata).

### 4.3 Clinical improvement and extubation (june 13 to june 21)

With metabolic parameters improving, sedation and analgesia were discontinued on June 13 after chemotherapy completion, allowing the patient to awaken; lactate levels decreased from 13.30 mmol/L on June 10 to 2.88 mmol/L. By June 18, lactate had further declined to 1.33 mmol/L, coinciding with removal of the pleural drainage tube and CRRT catheter. Respiratory function recovered progressively; on June 20, the patient exhibited stable spontaneous breathing, with arterial blood gas analysis showing pH 7.40 and PaO_2_ 95 mm Hg (FiO_2_ 40%), prompting extubation followed by sequential noninvasive ventilation. Noninvasive ventilation was discontinued on June 21, transitioning to low-flow nasal cannula oxygen (2 L/min).

### 4.4 Management of post-chemotherapy complications (late june)

Myelosuppression was addressed with transfusions of packed red blood cells and single-donor platelets for moderate anemia (hemoglobin 71 g/L on July 6) and thrombocytopenia, along with folic acid (5 mg three times daily) and iron supplementation to correct hematopoietic deficiencies.

For ICU-acquired weakness, physical examination on June 14 post-awakening revealed grade 1 muscle strength in all extremities (grade 1+ in the right lower limb); electromyography indicated multifocal peripheral nerve damage. Treatment included mecobalamin (Beijing Sunho Pharmaceutical Co., Ltd.) for neuroprotection, ambroxol combined with galantamine (Hubei Meilin) to enhance neuromuscular transmission, and early passive joint mobilization exercises in collaboration with rehabilitation services. By July 6, muscle strength had improved to grade 2-, enabling self-feeding without choking, and the nasogastric tube was removed.

### 4.5 Discharge status

**Figure 3:**
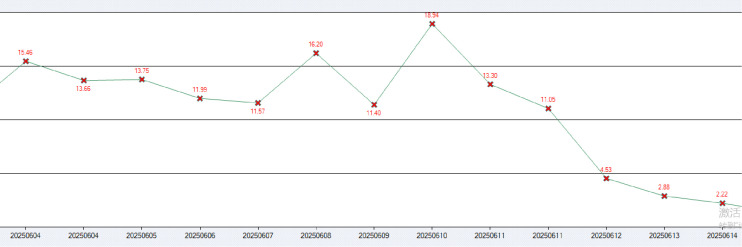
The dynamic trend of lactic acid changes during the treatment period Warburg effect and lactic acidosis.

The patient was transferred from the ICU to the general ward on June 27, 2025, for continued care. At discharge, he was afebrile with stable vital signs (temperature 36.5 °C, heart rate 80 beats per minute, respiratory rate 20 breaths per minute, blood pressure 144/88 mm Hg) and alert with appropriate responses. Lactate had normalized (0.6 mmol/L) (**Figure 3**), HBV-DNA decreased to 4.33 × 10³ IU/mL, platelets rose to 82 × 10^9^/L, and PCT was 0.244 ng/mL. Muscle strength was grade 2-, with independent oral intake and no aspiration; low-flow nasal cannula oxygen (2 L/min) was required.

Discharge instructions included continuation of oral entecavir (0.5 mg daily) for hepatitis B suppression, rivaroxaban (10 mg twice daily) for anticoagulation, and mecobalamin (0.5 mg three times daily) for neuroprotection. The patient was scheduled for the next chemotherapy cycle in 3 weeks, with regular follow-up monitoring of complete blood count, liver and kidney function, HBV-DNA, and lower extremity venous ultrasonography.

## 5 Ethical statement

This case report was conducted in strict accordance with the principles outlined in the Declaration of Helsinki and relevant medical ethics guidelines. The patient and family members were fully informed of the study’s objectives, data usage, and privacy protection measures, and provided voluntary written informed consent. Following review by the Institutional Review Board of the First Affiliated Hospital of Guangzhou Medical University, no ethical concerns were identified.

## 6 Literature review

Earlier studies have long recognized malignancy-associated type B lactic acidosis as a life-threatening metabolic complication, particularly in aggressive hematologic malignancies, with survival largely dependent on timely cytoreductive therapy (13, 15). The conceptual framework of cancer metabolic reprogramming provides biological plausibility for these bedside observations, in that tumors rewire nutrient acquisition and metabolism to meet bioenergetic, biosynthetic, and redox demands—often at the cost of increased lactate generation and microenvironmental remodeling (16). These landmark reports established the fundamental pathophysiological basis linking excessive tumor-driven lactate production to impaired systemic clearance and adverse outcomes. However, evolving intensive care strategies, advances in chemotherapy and targeted therapies, and improved supportive management warrant re-evaluation of contemporary cases. Building upon this foundation, we therefore focused our review on recent case reports to reflect current diagnostic approaches, organ support strategies, and treatment timing in modern clinical practice.

Malignancy-associated hyperlactatemia mediated by the Warburg effect (aberrant activation of aerobic glycolysis) represents a rare yet potent predictor of poor prognosis in patients with type B lactic acidosis, particularly those with hematologic malignancies or highly aggressive solid tumors. To delineate the clinical patterns, diagnostic challenges, and prognostic factors in this population, we systematically searched PubMed, Embase, and Cureus databases from January 2021 to May 2025 for case reports on the “Warburg effect” and “malignancy-associated hyperlactatemia, “ using keywords such as “Warburg effect, “ “malignancy-associated lactic acidosis, “ “type B lactic acidosis, “ and “non-shock lactic acidosis.” Ultimately, 18 eligible case reports were included (7, 14, 17–30), encompassing 12 patients with hematologic malignancies and 6 with solid tumors; key clinical features and treatment outcomes are summarized(**Table 1**).

**Table 1:** Core clinical characteristics of 18 patients with malignancy-associated Warburg effect and lactic acidosis.

Case	Hematologic tumor (yes or no)		Chemotherapy (yes or no)		Prognosis (improve or death)
Ziegler 2021 (17)	Yes		Yes		Death
Cao 2023 (14)	Yes		Yes		Death
Patel 2024 (18)	Yes		Yes		Death
Khanal 2024 (19)	Yes		Yes		Death
Sanivarapu 2022 (20)	Yes		Yes		Improve
Nzenwa 2023 (21)	Yes		Yes		Improve
Looyens 2021 (22)	Yes		Yes		Improve
Sedarous 2021 (23)	Yes			No	Death
Yamazaki 2025 (24)	Yes			No	Death
Moen 2023 (25)	Yes			No	Death
Kliebhan 2022 (26)	Yes			No	Death
Wang 2022 (7)	Yes			No	Death
Tang 2024		No	Yes		Improve
Karki 2023 (28)		No		No	Death
Rudy 2024 (29)		No		No	Death
Cheleng 2024 (30)		No		No	Death
Aly 2025 (31)		No		No	Death
Yokokawa 2025 (32)		No		No	Death

Regarding tumor type distribution, hematologic malignancies accounted for 66.7% (12/18) of cases, predominantly aggressive lymphomas, including diffuse large B-cell lymphoma (DLBCL) in 4 cases (7, 14, 20, 23), mantle cell lymphoma (MCL) in 3 cases (18, 21, 26), Burkitt lymphoma in 2 cases (19, 22), B-cell hepatosplenic lymphoma in 1 case (25), multiple myeloma in 1 case (17), and NK/T-cell lymphoma in 1 case (24). Solid tumors comprised 33.3% (6/18), involving colorectal cancer in 2 cases (31, 32), esophageal cancer in 1 case (28), cervical neuroendocrine carcinoma in 1 case (27), retroperitoneal leiomyosarcoma in 1 case (29), and cecal signet-ring cell carcinoma in 1 case (32). Notably, peak lactate levels were significantly higher in hematologic malignancies compared with solid tumors (median 15.4 mmol/L vs 7.5 mmol/L, *P* < 0.05). For instance, Khanal et al. (19) described a 69-year-old woman with Burkitt lymphoma whose lactate exceeded 15 mmol/L, accompanied by severe hypoglycemia (blood glucose declining from 70 mg/dL to 30 mg/dL); EPOCH chemotherapy normalized lactate within 48 hours, though she ultimately succumbed to neutropenic fever and sepsis post-chemotherapy. In contrast, among solid tumors, only Aly et al. (31) reported a 67-year-old man with advanced colorectal cancer reaching 15.4 mmol/L, with the remaining 5 cases below 10 mmol/L. This disparity may reflect the higher proliferative rates (e.g., Ki-67 index of 80% in an MCL case reported by Kliebhan et al. (26)) and systemic infiltration in hematologic malignancies; Yamazaki et al. (24) observed a similar pattern in NK/T-cell lymphoma, where extensive hepatic involvement tripled initial lactate levels, reinforcing the link between tumor burden and Warburg effect intensity.

The association between treatment regimens and prognosis emerged as a central finding. Among the 18 patients, 8 received regular antineoplastic therapy (defined as ≥1 full cycle at target doses without interruption), while 10 did not (including patient refusal, intolerance due to severe organ failure, or supportive care only). In the regular treatment group, 4 achieved clinical improvement (lactate normalization and radiographic tumor burden reduction), and 4 died. Looyens et al. (22) detailed a 60-year-old man with post-transplant Burkitt lymphoma complicated by peritoneal metastases and hyperlactatemia (7.4 mmol/L); R-CHOP chemotherapy combined with abdominal abscess drainage reduced lactate to 2.2 mmol/L within 1 week, with PET-CT confirming complete remission, enabling discharge after 5 months of hospitalization. Nzenwa et al. (21) reported a 66-year-old man with MCL (peak lactate 7.0 mmol/L) who normalized lactate within 3 days and recovered renal function following rituximab plus rasburicase for tumor lysis syndrome prevention, leading to successful discharge. Deaths in this group stemmed from chemotherapy complications intertwined with the primary malignancy; for example, Cao et al. (14) described a 76-year-old woman with DLBCL who, despite R-CHOP and continuous venovenous hemofiltration (CVVH) correcting hyperlactatemia, died from tumor lysis syndrome-induced septic shock. Sanivarapu et al. (20) noted a 48-year-old man with DLBCL whose lactate fell from 16 mmol/L to 3.5 mmol/L post-chemotherapy but succumbed to respiratory failure from progressive malignant pleural effusions, highlighting that while regular therapy effectively ameliorates metabolic disturbances, mitigating chemotherapy-related complications is equally vital. Prognosis in the non-regular treatment group was dismal, with all 10 patients dying within 7–21 days of admission and unrelenting lactate elevation. Cheleng et al. (30) reported a 52-year-old woman with metastatic colon cancer who refused chemotherapy and underwent only left hemicolectomy; postoperative lactate rose from 3.6 mmol/L to 6.5 mmol/L, culminating in coma from brain metastases and death. Rudy et al. (29) described a 69-year-old man with retroperitoneal leiomyosarcoma (ECOG performance status 4) intolerant to chemotherapy, receiving supportive care alone; peak lactate reached 20 mmol/L, leading to multiorgan failure and death. These cases underscore that tumor-directed therapy is pivotal for reversing Warburg effect-associated hyperlactatemia, with uniformly poor outcomes in its absence.

Contemporary clinical practice reveals ongoing controversies in managing these patients, particularly regarding supportive care strategies. Among the 18 cases, 6 involved bicarbonate infusion, 4 utilized CRRT/hemodialysis, and 2 included thiamine supplementation, with variable efficacy. Yokokawa et al. (32) reported a 66-year-old woman with cecal signet-ring cell carcinoma (lactate 7.5 mmol/L) whose arterial pH transiently improved from 7.38 to 7.45 with bicarbonate, yet lactate remained unchanged. In contrast, Tang et al. (27) described a 59-year-old woman with cervical neuroendocrine carcinoma (lactate 11.2 mmol/L) where bicarbonate combined with chemotherapy reduced lactate to 5.9 mmol/L within 48 hours, indicating that bicarbonate merely temporizes acid-base imbalances and cannot independently curb Warburg-mediated lactate production without concomitant antineoplastic therapy. Similar limitations apply to CRRT/hemodialysis; Patel et al. (18) detailed a woman with MCL (lactate 16.6 mmol/L and acute kidney injury) whose lactate fell to 2.8 mmol/L over 5 days with hemodialysis plus rituximab and acalabrutinib, alongside renal recovery. However, Moen et al. (25) reported a 70-year-old man with B-cell hepatosplenic lymphoma who, despite CRRT, maintained lactate >20 mmol/L without timely chemotherapy, dying 11 days post-splenectomy—emphasizing that renal replacement therapy suits those with concurrent organ failure but cannot supplant tumor-specific interventions.

Challenges in chemotherapy timing and patient selection also merit attention. In 3 cases, initial lactate >15 mmol/L was deemed a “contraindication” to chemotherapy, delaying treatment and worsening prognosis. Wang et al. (7) described a DLBCL patient with lactate at 30 mmol/L where the team prioritized metabolic correction before R-CHOP, initiating only at 18 mmol/L; rapid tumor progression led to death within 1 week. Conversely, Khanal et al. (19) promptly started EPOCH for Burkitt lymphoma despite lactate >15 mmol/L, achieving rapid control—contrasting outcomes that suggest early chemotherapy is warranted absent irreversible organ failure (e.g., severe hepatic failure or irreversible brain injury). For frail patients, Tang et al. (27) employed a “dose-reduced” regimen (etoposide 100 mg on days 1, 3, 4), mitigating toxicity while controlling lactate (declining to 2.2 mmol/L), enabling discharge for palliative care and offering a viable approach for critically ill individuals.

Drawing from this analysis of 18 cases of Warburg effect-associated malignancy with hyperlactatemia, we infer that the phenomenon is more pronounced in hematologic malignancies (especially aggressive lymphomas), with peak lactate typically exceeding that in solid tumors; regular antineoplastic therapy is crucial for prognosis improvement, with 100% mortality in untreated patients, though chemotherapy complications interacting with the primary tumor drive fatalities; supportive measures (e.g., bicarbonate infusion, CRRT) serve adjunctively and cannot replace targeted therapy. Future prospective studies are needed to refine strategies, exploring criteria integrating “lactate levels + tumor proliferative activity” to optimize chemotherapy initiation for these critically ill patients.

## 7 Discussion

Recent studies have emphasized that lactate is not merely a byproduct of glycolysis but a key signaling metabolite within the tumor microenvironment (TME), capable of promoting tumor progression through multiple mechanisms. First, lactate accumulation and acidification of the TME suppress the proliferation, cytotoxic activity, and cytokine production of effector T cells and natural killer cells, while promoting the differentiation and maintenance of immunosuppressive cell populations such as regulatory T cells and M2-like macrophages, thereby establishing a state of “metabolic immunosuppression” and impairing antitumor immunity (33, 34). Second, lactate facilitates tumor growth and metastasis by regulating cancer cell migration, angiogenesis, and therapeutic resistance, and has been closely associated with unfavorable clinical outcomes, suggesting that lactate itself may function as an active oncometabolite driving disease progression (35, 36). In addition, lactate acts as a metabolic–epigenetic hub by inducing protein lactylation and reprogramming transcriptional profiles in both tumor and immune cells, further amplifying immunosuppressive and metabolic reprogramming effects and providing potential therapeutic targets for disrupting lactate production, transport, and utilization (37).

In this case, the patient with ALK-positive anaplastic large cell lymphoma (ALK+ ALCL) presented with fever and developed persistent hyperlactatemia without evidence of shock, accompanied by type II respiratory failure, hepatic impairment, and electrolyte disturbances. Hemodynamics remained stable throughout admission and ICU stay (systolic blood pressure 144–161 mm Hg), with arterial partial pressure of oxygen within normal or acceptable ranges, suggesting that type A mechanisms (related to hypoperfusion or hypoxia) were not predominant. Given the oncologic context and laboratory features (elevated LDH), this phenotype aligns with the spectrum of clinical Warburg effect (CWE) or malignancy-associated type B lactic acidosis: enhanced aerobic glycolysis in tumor cells drives lactate production even under adequate tissue oxygenation, with further accumulation amid reduced hepatic (accounting for ~60% of clearance) or renal (~30%) function (12, 38, 39). Importantly, CWE is not an exclusionary diagnosis; it should be established only after thorough evaluation to rule out type A etiologies, drugs/toxins, severe hepatorenal failure, or other metabolic causes (40).

Consistent with prior studies (41, 42), lactate trajectory in this case closely paralleled oncologic interventions, with rapid and sustained decline following initiation of an etoposide-containing CHOP variant (CHOEP/ECHOP, i.e., CHOP plus etoposide)—a “metabolic response” temporally linked to tumor burden control. Chaba et al. (12) reported that CWE patients typically exhibit higher tumor burden, more frequent bone or central nervous system involvement, and substantially elevated 1-year mortality risk (multivariable HR = 3.89, 95% CI 2.13-7.02). Thus, rather than targeting lactate reduction per se, a more rational approach prioritizes etiology-directed tumor control, with internal milieu interventions serving as bridges to enhance tolerability.

Regarding organ support and timing, chemotherapy was promptly instituted post-diagnosis, with concurrent CRRT to address severe metabolic acidosis and electrolyte imbalances. Notably, CRRT offers limited direct value for lactate clearance and has not been shown to improve long-term survival; routine use is inadvisable absent clear renal replacement indications, positioning it more as a facilitator of metabolic and electrolyte stability to enable chemotherapy (1). Extrapolation should be cautious, as evidence for CRRT’s protective role against chemotherapy toxicity is inconsistent; dosing must be individualized based on pharmacokinetics and RRT modality, rather than assuming clearance of toxic metabolites. The concomitant hepatic dysfunction in this case may have diminished lactate clearance, a phenomenon observed in sepsis and critical illness cohorts (43, 44), though its relative contribution is challenging to disentangle from excess production—favoring parallel rather than singular causal reasoning in clinical judgment.

For regimen selection, evidence increasingly favors shifting from conventional CHOP/CHOEP to BV-CHP (brentuximab vedotin plus CHP) as first-line therapy. The 5-year update from the ECHELON-2 trial (45) demonstrated sustained PFS and OS benefits with BV-CHP over CHOP in peripheral T-cell lymphomas (including systemic ALCL). However, in the ICU setting, constrained by drug availability, infection risks, organ function, and hematologic tolerance, CHOEP/ECHOP remains a pragmatic, rapidly deployable first-line option, with multidisciplinary teams balancing early tumor control against tolerability. ALK inhibitors (e.g., crizotinib, brigatinib) have limited frontline evidence in adult ALK+ ALCL, primarily reserved for relapsed/refractory disease or select subgroups in trials and real-world settings (46–48); their potential for faster hyperlactatemia reversal lacks systematic data and warrants cautious use in monitored, individualized protocols or clinical trials.

Complication management in this case addressed post-chemotherapy myelosuppression and ICU-acquired weakness (ICU-AW) risk. Myelosuppression was managed with infection prophylaxis, G-CSF, targeted transfusions, and nutritional support. Pharmacologic platelet elevation and neurotrophic therapies currently lack high-level evidence, emphasizing early mobilization and rehabilitation as core strategies. Recent studies (49, 50) indicate that early active exercise/rehabilitation reduces ICU-AW incidence and enhances functional outcomes. Additionally, as an HBsAg-positive patient, prophylactic antiviral therapy (entecavir) with serial HBV-DNA monitoring aligned with consensus for oncologic treatment in HBV carriers. ASCO guidelines and recent investigations recommend preemptive antivirals and vigilant follow-up to mitigate reactivation risk (51, 52).

## 8 Conclusion

Overall, this case underscores that persistent hyperlactatemia amid hemodynamic stability warrants CWE as a key differential consideration. Therapeutically, etiology-directed tumor control offers a sustainable path to metabolic crisis resolution, with CRRT and other supports bridging to chemotherapy tolerability; regimen choices must balance evidence-based standards (e.g., BV-CHP) against critical care constraints. Future prospective studies are recommended to systematically evaluate such cases, exploring integration of small-molecule ALK inhibitors with chemotherapy in adult ALK+ ALCL.

## Data Availability

The original contributions presented in the study are included in the article/**Supplementary Material**. Further inquiries can be directed to the corresponding author.

## Supplementary material

Supplementary Figure 1 diagnostic and treatment pathway timeline.
