# Supplementary figures and images for: Persistent lactic acidosis in ALK-positive anaplastic large cell lymphoma: a case report and literature review
Source: Front Oncol. 2026 Feb 27;16:1744202. doi: 10.3389/fonc.2026.1744202 (PMC12982027; doi:10.3389/fonc.2026.1744202)

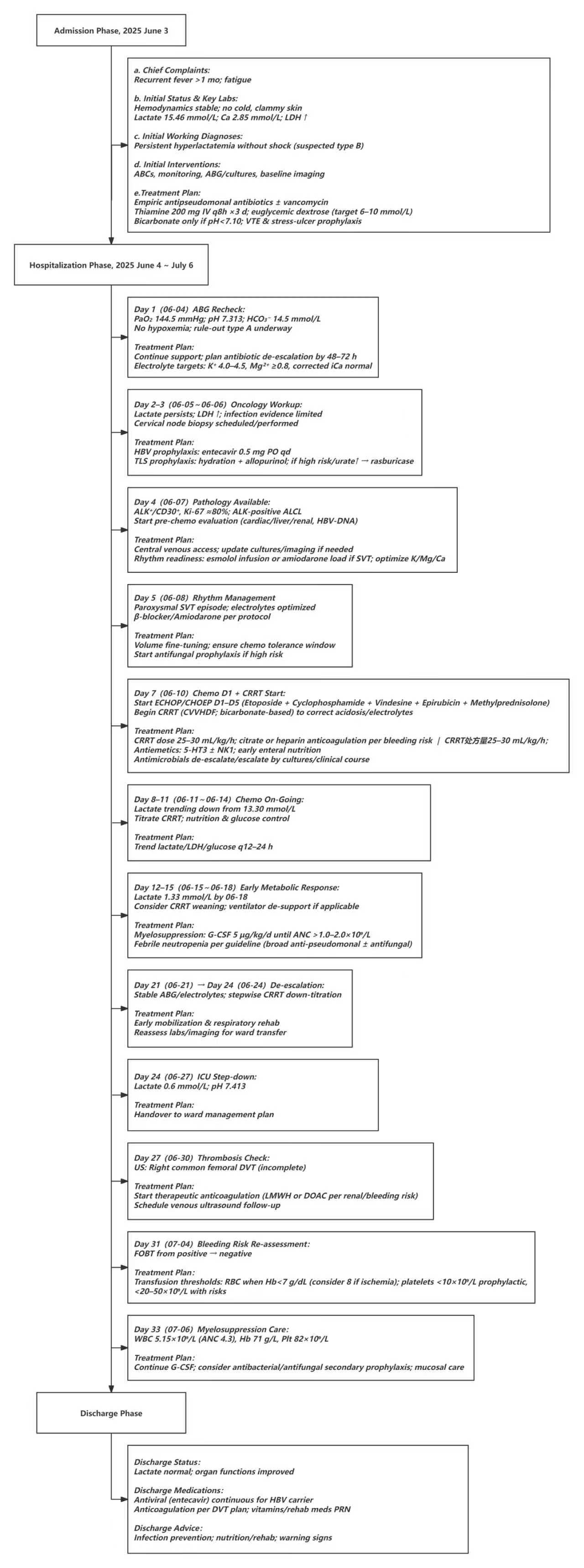

Supplement: Supplementary Figure 1 — diagnostic and treatment pathway timeline. [file DataSheet1.zip › Figure 1.jpg]

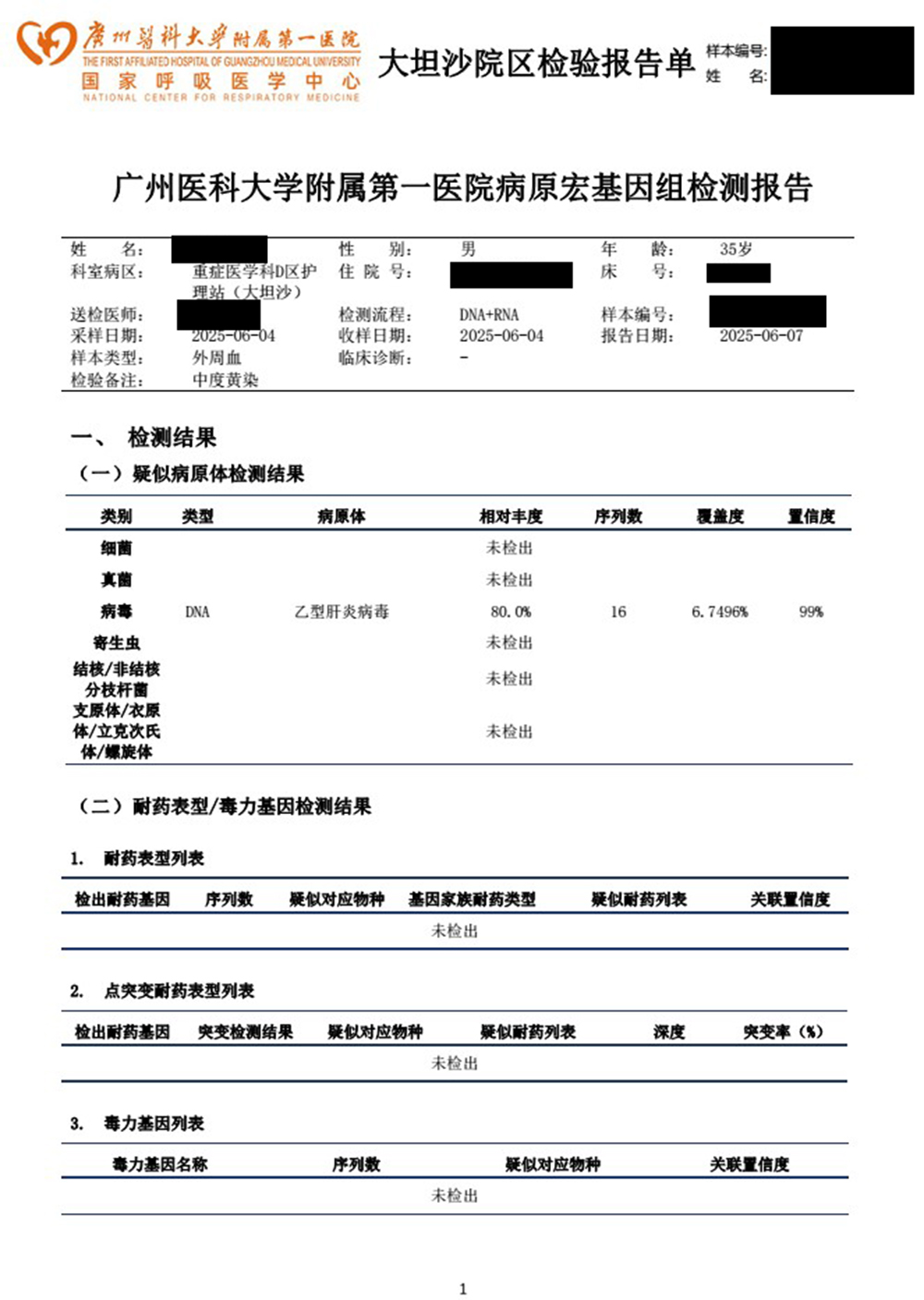

Supplement: Supplementary Figure 1 — diagnostic and treatment pathway timeline. [file DataSheet1.zip › NGS1.jpg]

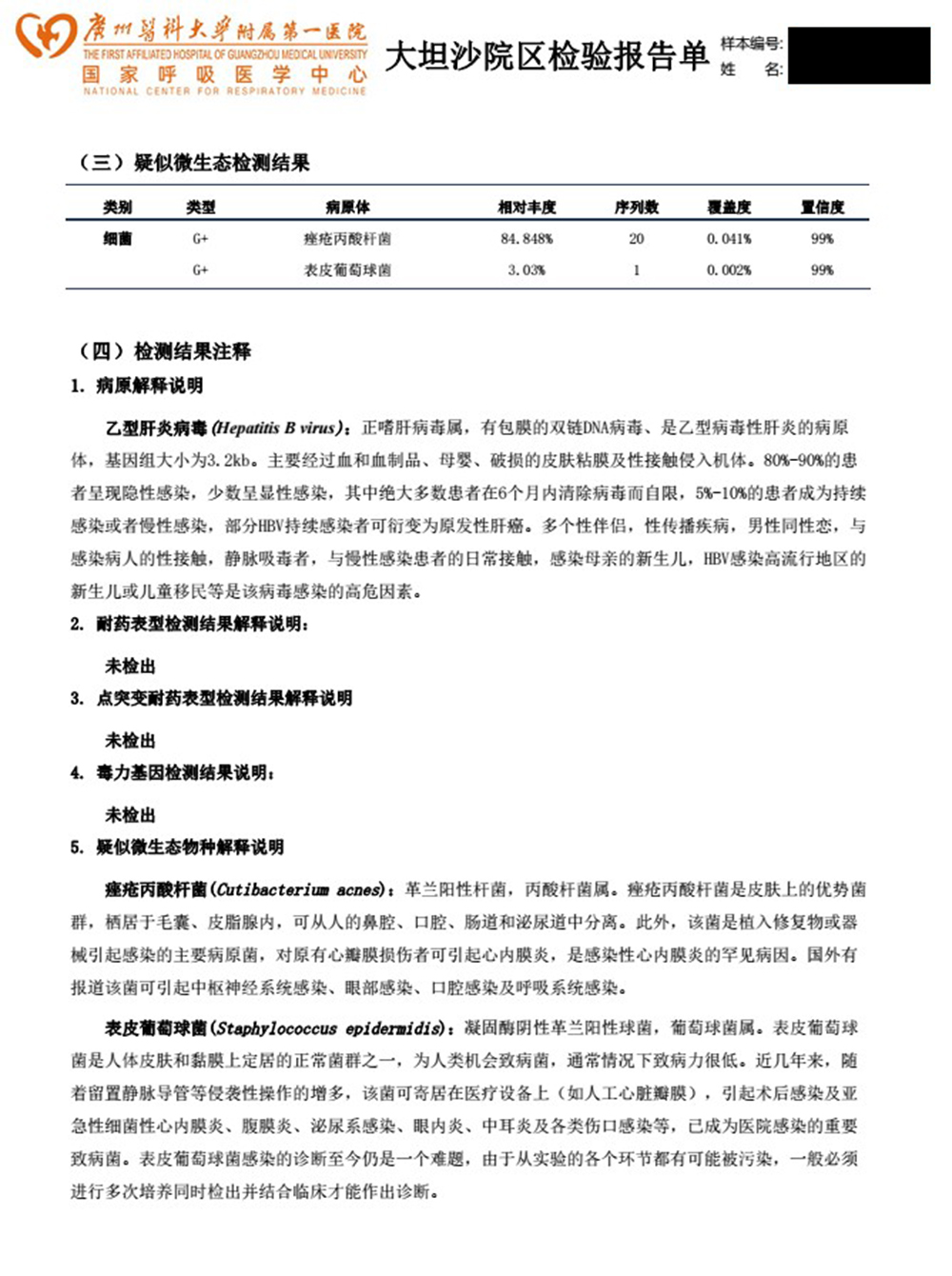

Supplement: Supplementary Figure 1 — diagnostic and treatment pathway timeline. [file DataSheet1.zip › NGS2.jpg]

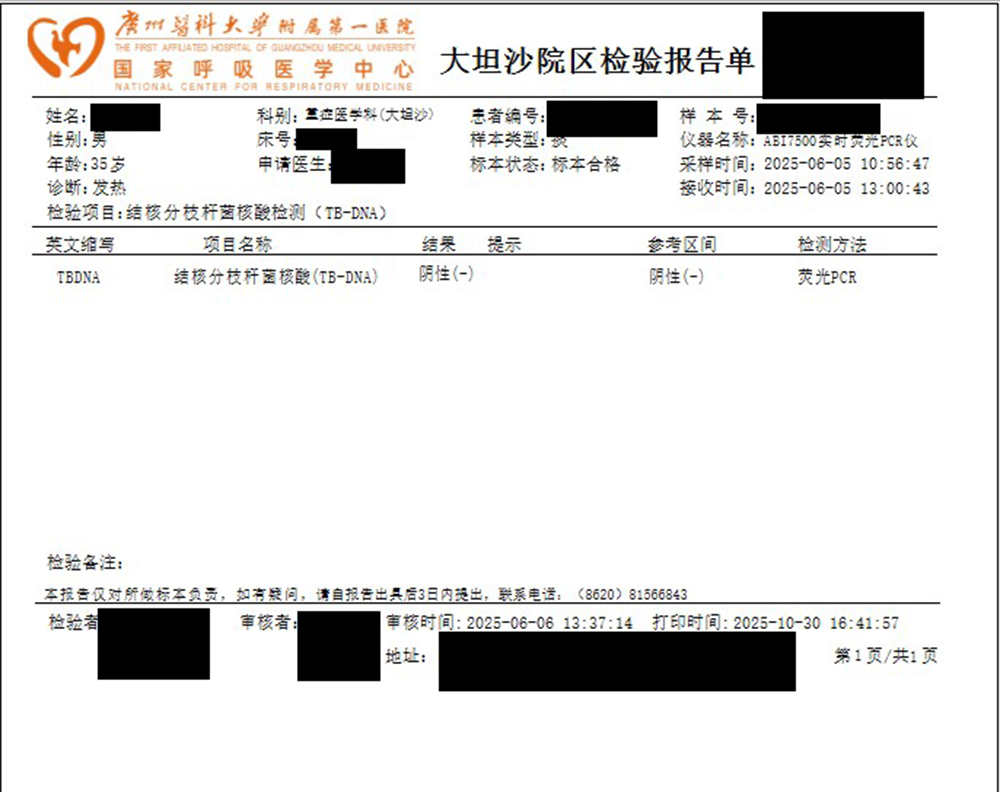

Supplement: Supplementary Figure 1 — diagnostic and treatment pathway timeline. [file DataSheet1.zip › TB-DNA.jpg]

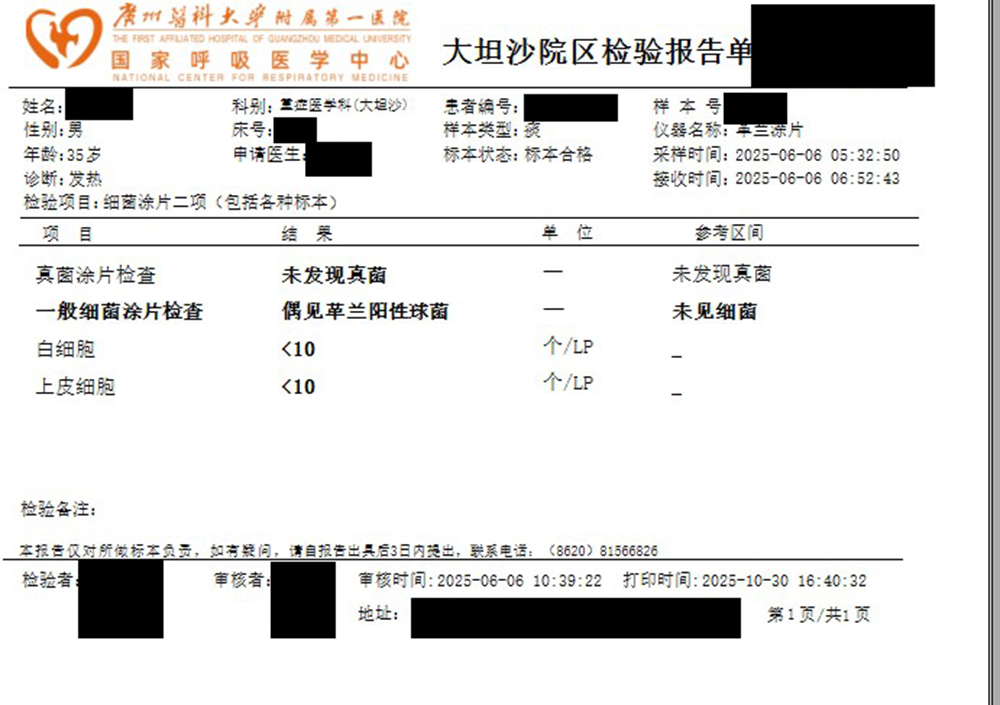

Supplement: Supplementary Figure 1 — diagnostic and treatment pathway timeline. [file DataSheet1.zip › 一般细菌涂片.jpg]

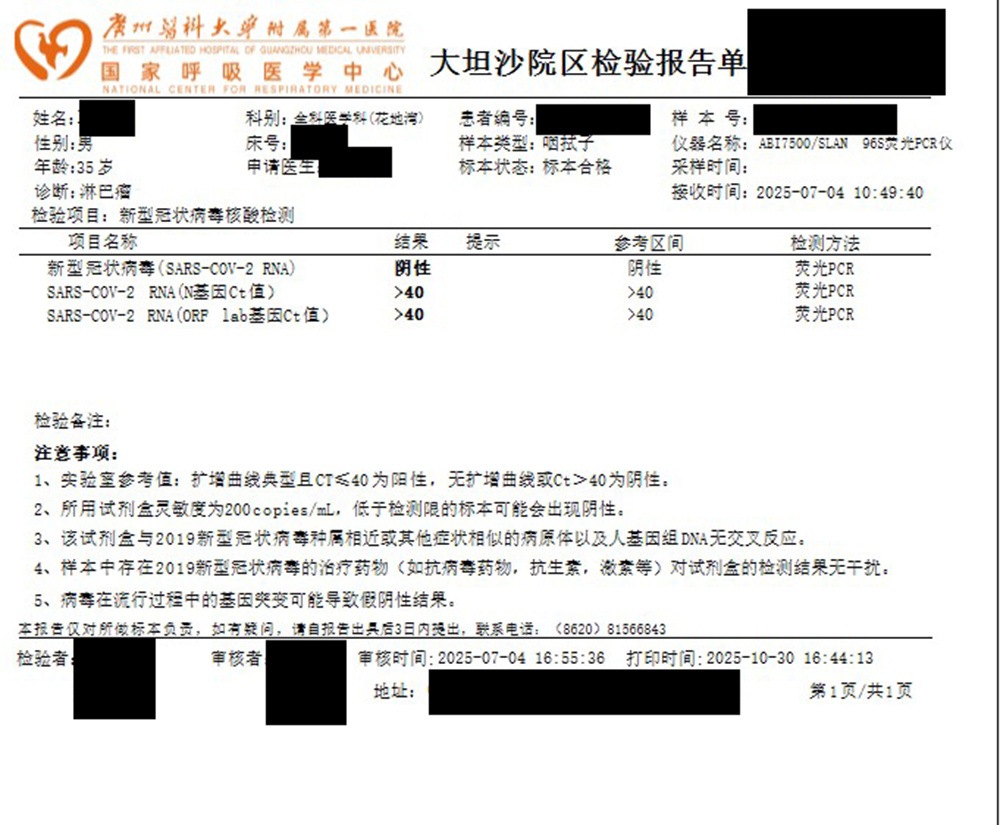

Supplement: Supplementary Figure 1 — diagnostic and treatment pathway timeline. [file DataSheet1.zip › 新冠病毒核酸.jpg]

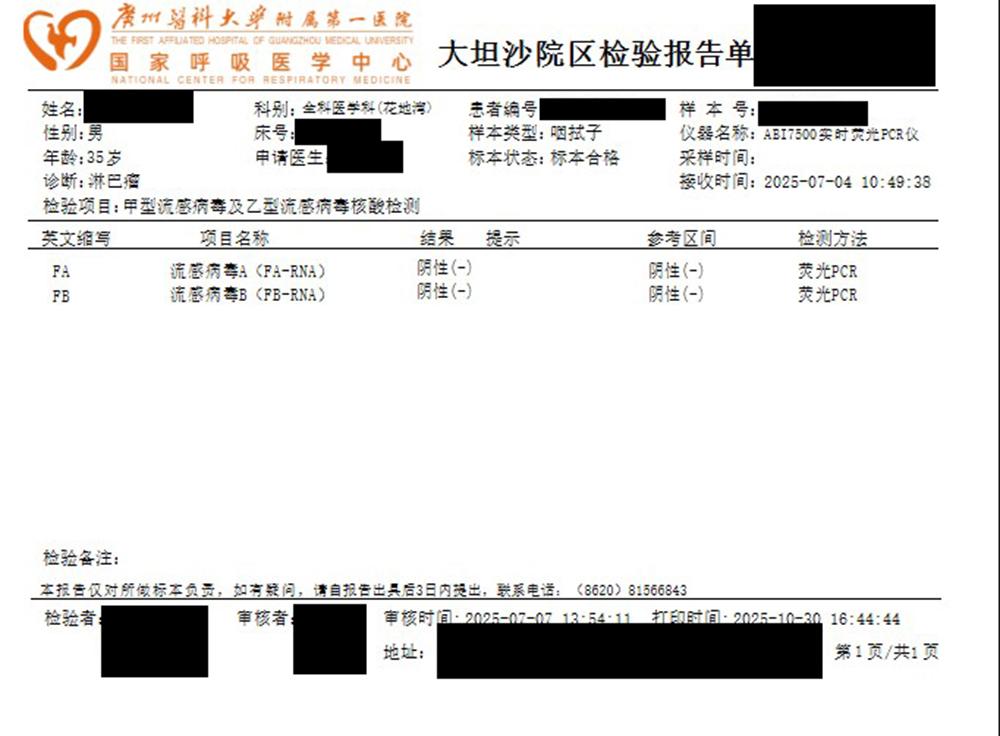

Supplement: Supplementary Figure 1 — diagnostic and treatment pathway timeline. [file DataSheet1.zip › 甲乙流核酸.jpg]

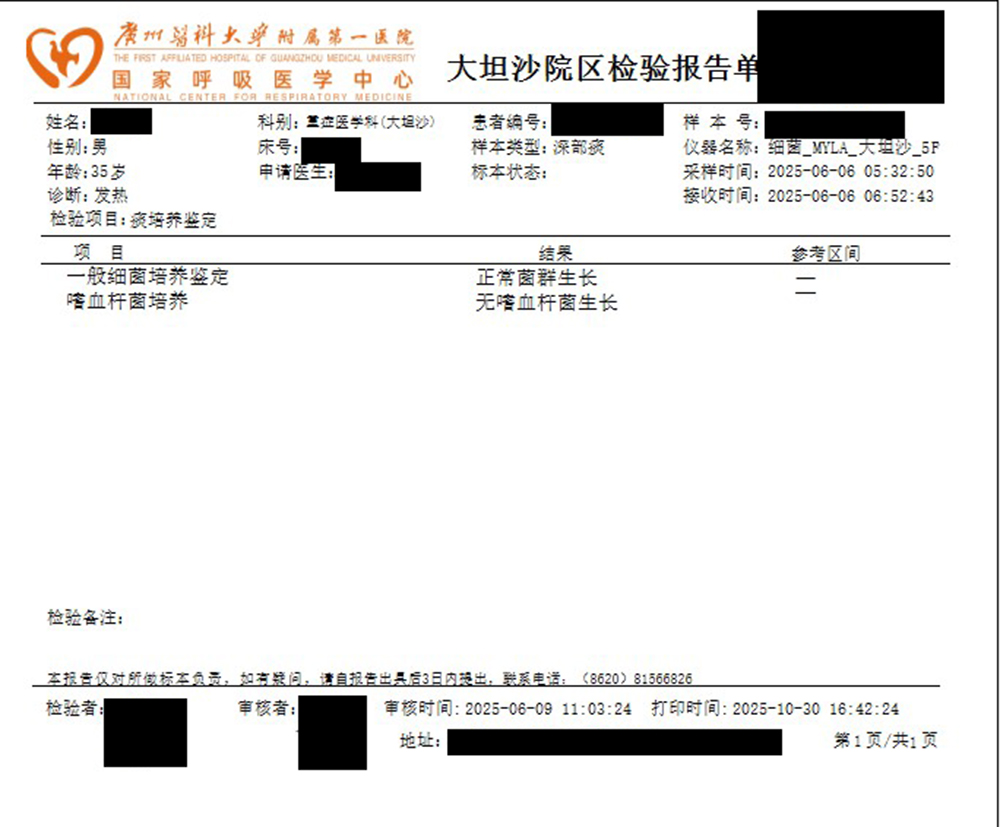

Supplement: Supplementary Figure 1 — diagnostic and treatment pathway timeline. [file DataSheet1.zip › 痰培养.jpg]

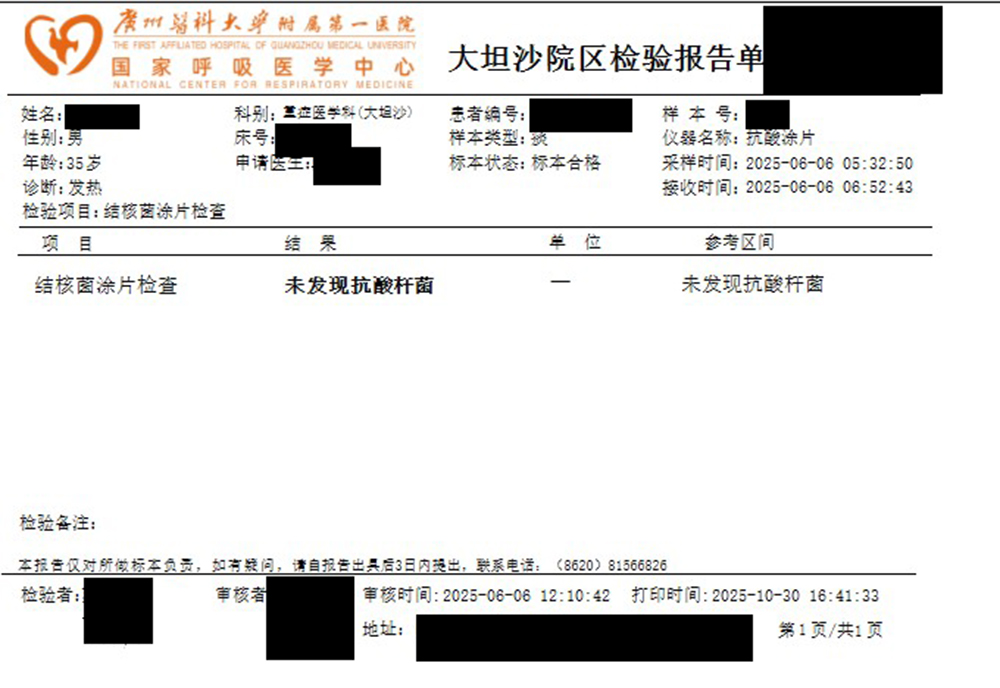

Supplement: Supplementary Figure 1 — diagnostic and treatment pathway timeline. [file DataSheet1.zip › 痰结核涂片.jpg]

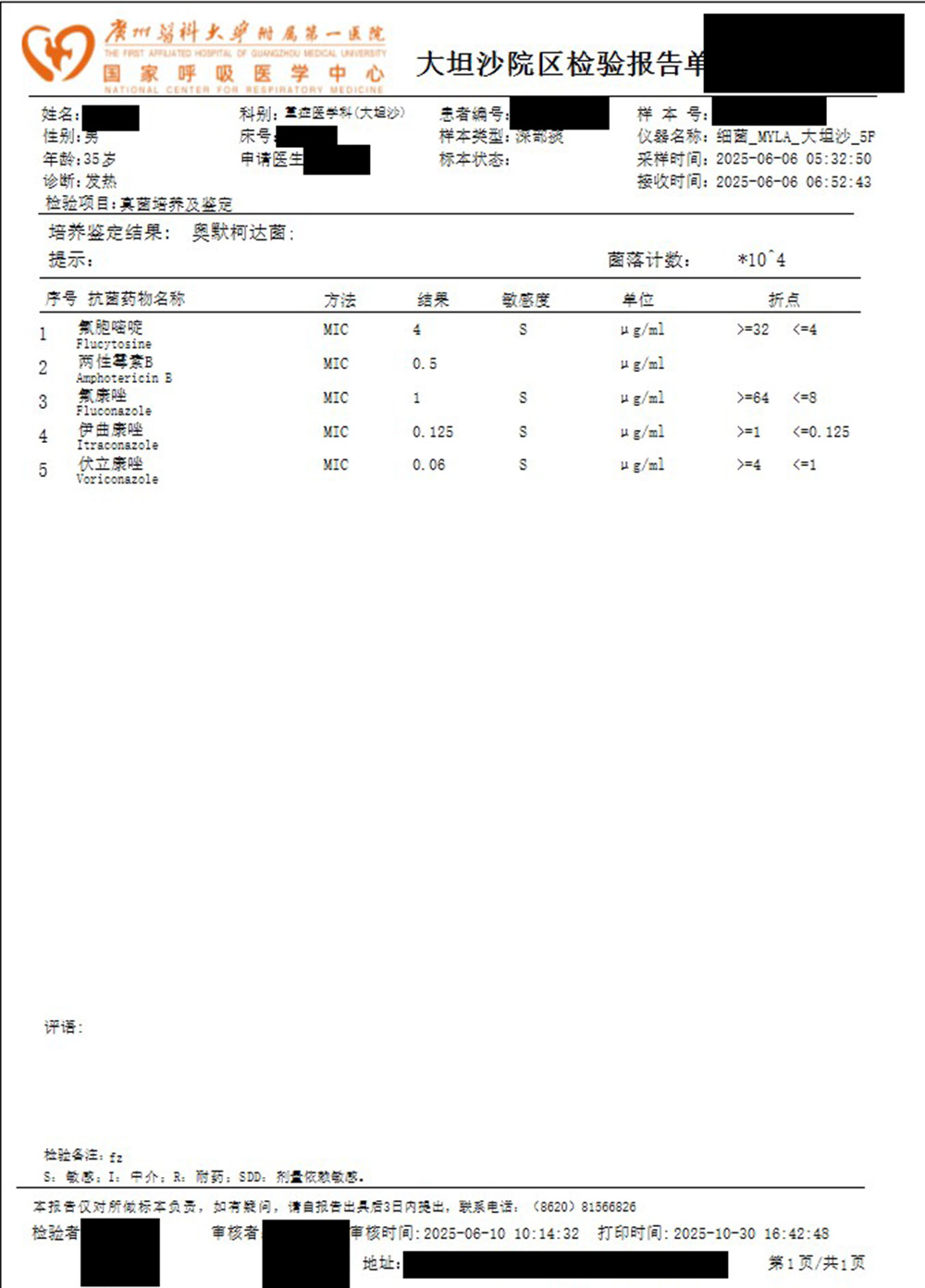

Supplement: Supplementary Figure 1 — diagnostic and treatment pathway timeline. [file DataSheet1.zip › 真菌培养及鉴定.jpg]
